# Vaccines and Vaccine Adjuvants for Infectious Diseases and Autoimmune Diseases

**DOI:** 10.3390/vaccines11020202

**Published:** 2023-01-17

**Authors:** Kutty Selva Nandakumar

**Affiliations:** Department of Environmental and Biosciences, School of Business, Innovation and Sustainability, Halmstad University, 30118 Halmstad, Sweden; kutty-selva.nandakumar@hh.se

## 1. Microbes in Health and Disease

A dynamic association of specific microbiota during different stages of human life is well documented [1]. Interactions between such microbes and humans are not only involved in basic, beneficial biological processes, but also in preventing the development and/or progression of several chronic diseases such as infectious diseases, cancer, metabolic diseases, gastro-intestinal diseases, liver diseases, respiratory diseases, autoimmune diseases, cardiovascular diseases, psychiatric diseases, and so on, affecting various vital organs and physiological functions in the body. Microbes living on mucosal surfaces, such as oral, gastro-intestinal, reproductive, and respiratory tracts, as well as skin, are essential for our health, and dysbiosis of normal microbiota in these locations causes serious health complications [2].

## 2. Infectious Diseases

Infectious diseases are continuously emerging, and these require early diagnostic methods, therapeutic drugs, and vaccines. A plethora of infectious diseases caused by pathogenic viruses and bacteria, such as flu, COVID-19, yellow fever, dengue fever, Ebola, AIDS, Middle East respiratory syndrome (MERS), Lassa fever, chikungunya, cholera, typhoid fever, and many others, are increasingly affecting humans worldwide. Vaccines using live-attenuated or whole-inactivated pathogens, mRNA, DNA, recombinant vectors (non-replicating, live-attenuated) and proteins are used or being developed to combat these infectious diseases [3], and some of them require appropriate adjuvants to induce optimal immune responses. In addition to early development, large-scale manufacturing possibilities, logistics, technology transfer, costs, and regulatory approval are some of the other factors involved in developing and administering vaccines at a global level [4]. At the same time, constant monitoring of the individuals for a short and long duration after vaccine administration for vaccine efficacy, adverse effects (antibody-dependent enhancement, vaccine-associated enhanced respiratory disease, autoimmunity reactions, etc.), vaccine-induced immune responses, risk management, stability of vaccines during storage and transport, potency of vaccine adjuvants, booster requirements and interval also need to be adequately considered in a successful vaccine strategy. Similarly, surveillance of emerging changes in pathogens (adaptation, mutations, gene deletions, changes in the virulence factors, new variants, etc.) that could spread faster to cause an increased lethality and mortality is essential to successfully combat the infections. Most importantly, an increase in the world population, global travel, and destruction of the natural habitats of other species endangers the prevailing natural safety barriers for the transmission of infections, leading to the emergence of new and more dangerous pathogens with fast transmission capacities across the species at a global level, damaging the collective health of our society with severe economic consequences and sustainability issues. In this context, vaccination is the most effective method of preventing the infections’ associated morbidity and mortality, especially in older and at-risk populations. Age-related alterations in the immune system lead to decreased immune responses and clinical efficacy of vaccines in elderly populations [5]. Similarly, people with health complications, such as hypertension, diabetes, morbid obesity, liver disease, chronic heart patients, kidney disease, individuals with chronic respiratory or pulmonary issues, and immuno-compromised individuals such as AIDS patients, need vaccination to prevent severe disease development or death. Vaccines eliciting both cellular and humoral responses are essential for the successful eradication of infectious pathogens such as bacteria and viruses from the body. Alterations in the gut microbiota are reported to be associated with vaccine immunogenicity and adverse events [6], suggesting that vaccination-induced changes in our microbiota composition could also have an impact on our overall health. Interestingly, outer-membrane vesicles (OMV) derived from bacteria can be used as vaccines to prevent infections [7], as well as to protect against bone loss [8]. Genetic engineering technology is being used to improve and expand the usefulness of such OMV vaccines by reducing toxicity and, in expressing the crucial antigens, multiple antigenic variants and heterologous antigens. Thus, the usefulness and future perspectives of OMV-based vaccines are enormous [9].

## 3. Autoimmune Diseases

On the other hand, 3–5% of people worldwide are affected by autoimmunity, an inappropriate immune response against self-antigens, which results from complex interactions between certain genetic and environmental factors. Although autoimmune diseases are classified as T or B cell-driven diseases, in reality, several immune and non-immune cells secreting pro-inflammatory mediators—including autoantibodies, cytokines, chemokines, radicals, and enzymes—are involved in the destruction of the target tissue and perpetuation of autoimmune disease pathology. Microbes are key elements in developing and controlling our immune responses; therefore, infections might be involved in triggering an autoimmune response either in an antigen-specific or non-specific manner. For example, an association between pathogens (bacteria and viruses) and autoimmune diseases is well known [10,11,12]. Since all autoimmune conditions are associated with at least one type of infection, it would be interesting to investigate the protective effects of vaccinations against infectious diseases in some autoimmune conditions as well. At the same time, prevailing autoimmunity can also predispose to different infectious diseases [13]. For example, neutralizing autoantibodies to key molecules such as cytokines might promote ongoing infectious diseases or increases the risk for them. Several adverse responses, such as arthritis, vasculitis, encephalitis, neuropathy, and demyelination, have been reported after vaccination [14]. Although a causal relationship between vaccination and autoimmunity is difficult to establish, indirect evidence shows the existence of such a connection. Some of the possible mechanisms by which vaccines can trigger autoimmune responses could be molecular mimicry, epitope spreading, bystander activation, and polyclonal activation by antigens from the infectious agents; antigen protection and translocation to lymphoid organs, the activation of Toll-like receptors, dendritic cells, and macrophages, as well as the activation of cellular immune responses by vaccine adjuvants; or allergic reactions and overdose by preservatives and other vaccine components that might trigger immune responses, resulting in the initiation or perpetuation of autoimmune responses [14]. Of note, a pre-requisite for microbial products for the induction of disease manifestations in many animal models of several autoimmune diseases is well known.

Interestingly, a therapeutic vaccination to autoimmune diseases that can resolve or control inflammatory immune responses is an interesting area of research. The efficacy of therapeutic vaccination for ongoing arthritis with a collagen type II peptide complexed with MHC class II molecules was shown in earlier studies [15,16]. Therefore, antigen-specific tolerization and targeted delivery as a therapeutic strategy is crucial in controlling and/or resolving autoimmune diseases [17]. At the same time, the non-specific and sex-differential effects of vaccines should also be considered [18]. In this context, it is also of interest to discuss vaccination effects against infectious diseases in patients with autoimmune diseases and the precautions needed while vaccinating patients receiving therapeutic and immunosuppressive drugs. However, whether vaccines against autoimmune diseases affect susceptibility or severity to infectious diseases or not is still unclear.

## 4. Vaccine Adjuvants

Adjuvants boost the potency and longevity of vaccines but cause minimal adverse effects or their own long-lasting immune responses. Adding adjuvants to vaccines increases and prolongs specific immunity to the antigens that are present in the vaccines, thereby reducing the amount and number of required vaccinations. Therefore, properly formulated adjuvants are essential for increasing the potency of a vaccine. Although aluminium salts (Alum) capable of inducing a strong and long-lasting antibody response are traditionally used as adjuvants in human vaccines, they are poor inducers of T cell responses; thus, novel adjuvants and formulations are required. Pathogen-associated molecular patterns (PAMPs) are recognized by pathogen recognition receptors (PRRs), which bind microbial ligands to induce immune responses, and the PAMPs binding to Toll-like receptors are the basis of several adjuvants [19]. Moreover, components such as cytokines, bacterial toxins, and glycolipids altering antigen processing are used as adjuvants to induce appropriate immune responses. PRRs such as Toll-like receptors, RIG-I-like receptors, C-type lectin receptors, and NOD-like receptors recognize the viral nucleic acids and bacterial components present in the vaccine/adjuvant formulations and induce immune responses. After binding to their ligands, PRRs engage with multiple adaptor proteins to initiate the signal transduction cascades, resulting in the activation of protein kinases, regulatory factors, and transcription factors affecting the gene transcription and translation of several pro-inflammatory mediators that are involved in the clearance of invading pathogens. Therefore, several compounds and mechanisms are used in adjuvant formulations to increase the immunological responses. For example, the development of long-lasting antigen depots, enhanced antigen presentation by activated DCs, and increased CD8^+^ and CD4^+^ T cell responses are considered while selecting appropriate adjuvants [20]. In this context, several natural and stimuli-responsive synthetic polymers are being considered for their adjuvant potential: properties such as polymer composition, chain length and format, charge density and ratio, balance between hydrophobicity and hydrophilicity, biocompatibility, biodegradability, easy synthesis and purification, and non-toxic properties determine their use as adjuvants [21].

Therefore, this article collection aims to increase our knowledge on vaccines for several infectious diseases, as well as issues surrounding their widespread use in the general and at-risk population; on emerging concepts for developing new vaccines to autoimmune diseases; and on the development of potential vaccine adjuvants to augment the potency and long-term use of such vaccines. Thus, we invite original research articles, reviews, viewpoints, and perspectives emphasizing the contribution of vaccines in controlling infectious diseases and autoimmune diseases, as well as the development and characterization of potential vaccine adjuvants. Articles describing issues related to vaccine development, administration, efficacy, adverse reactions, monitoring immune and clinical responses to vaccines, and identifying genetic and environmental factors that could potentially affect vaccine potency, are also most welcome. In the future, these studies may lead to the development of new vaccines, adjuvants and formulations, and delivery systems, with reduced toxicity and cost of production and delivery for targeted populations.
